# The Effects of Intramuscular Naloxone Dose on Mu Receptor Displacement of Carfentanil in Rhesus Monkeys

**DOI:** 10.3390/molecules25061360

**Published:** 2020-03-17

**Authors:** Peter J. H. Scott, Robert A. Koeppe, Xia Shao, Melissa E. Rodnick, Alexandra R. Sowa, Bradford D. Henderson, Jenelle Stauff, Phillip S. Sherman, Janna Arteaga, Dennis J. Carlo, Ronald B. Moss

**Affiliations:** 1 Department of Radiology, University of Michigan, Ann Arbor, MI 48105, USA; koeppe@med.umich.edu (R.A.K.); xshao@umich.edu (X.S.); topperm@umich.edu (M.E.R.); sowa@umich.edu (A.R.S.); bkhend@umich.edu (B.D.H.); jrstauff@umich.edu (J.S.); psherman@umich.edu (P.S.S.); jannaa@umich.edu (J.A.); 2 Adamis Pharmaceuticals, 11682 El Camino Real, Suite # 300, San Diego, CA 92130, USA; Dcarlo@adamispharma.com

**Keywords:** opioid, naloxone, overdose, fentanyl, carfentanil, [^11^C]carfentanil, positron emission tomography, receptor occupancy, pharmacokinetics

## Abstract

Naloxone (NLX) is a mu receptor antagonist used to treat acute opioid overdoses. Currently approved doses of naloxone to treat opioid overdoses are 4 mg intranasal (IN) and 2 mg intramuscular (IM). However, higher mu receptor occupancy (RO) may be required to treat overdoses due to more potent synthetic opioids such as fentanyl and carfentanil that have entered the illicit drug market recently. To address this need, a higher dose of NLX has been investigated in a 5 mg IM formulation called ZIMHI but, while the effects of intravenous (IV) and IN administration of NLX on the opioid mu receptor occupancy (RO) have been studied, comparatively little is known about RO for IM administration of NLX. The goal of this study was to examine the effect of IM dosing of NLX on mu RO in rhesus macaques using [^11^C]carfentanil positron emission tomography (PET) imaging. The lowest dose of NLX (0.06 mg/kg) approximated 51% RO. Higher doses of NLX (0.14 mg/kg, 0.28 mg/kg) resulted in higher mu RO of 70% and 75%, respectively. Plasma levels were 4.6 ng/mL, 16.8 ng/mL, and 43.4 ng/mL for the three IM doses, and a significant correlation between percent RO and plasma NLX level was observed (r = 0.80). These results suggest that higher doses of IM NLX result in higher mu RO and could be useful in combating overdoses resulting from potent synthetic opioids.

## 1. Introduction

Mortality from drug overdoses has reached epidemic proportions in the U.S. (>70,000 in 2017), exceeding the number of yearly deaths during the peak of the AIDS epidemic [1]. One of the key drivers of the overdoses has been the abuse of synthetic opioids [2]. Naloxone (NLX) is the first line of treatment and an effective countermeasure in the event of an opioid overdose because, as a mu receptor antagonist, it is capable of rapid reversal of opioid toxicity [3]. Currently approved doses of NLX include 4 mg intranasal (IN) and 2 mg intramuscular (IM) [4] but, to date, there have been numerous reports suggesting that multiple doses of NLX may be required for successful reversal of opioid toxicity, especially when treating overdoses due to more potent synthetic opioids such as fentanyl and carfentanil that have entered the illicit drug market recently [5,6,7,8,9]. To address this issue, we previously reported that a higher dose of intramuscular naloxone (5 mg ZIMHI) has greater systemic exposure compared to the current community dose of naloxone (2 mg intramuscular and 4 mg intranasal) [10]. However, while the effects of intravenous (IV) and IN administration of NLX on mu opioid receptor occupancy (RO) have been studied, comparatively little is known about RO resulting from IM administration [11,12]. The goal of this study was, therefore, to examine the effects of IM NLX on mu RO in rhesus macaques. Using [^11^C]carfentanil ([^11^C]CFN, [13]), we employed preclinical positron emission tomography (PET) imaging to investigate RO of NLX administered by IM injection at three different doses in two mature rhesus monkeys, and we compared our imaging findings with the plasma levels of NLX in the monkeys determined by quantitative LC-MS/MS.

## 2. Results

**Figure 1:**
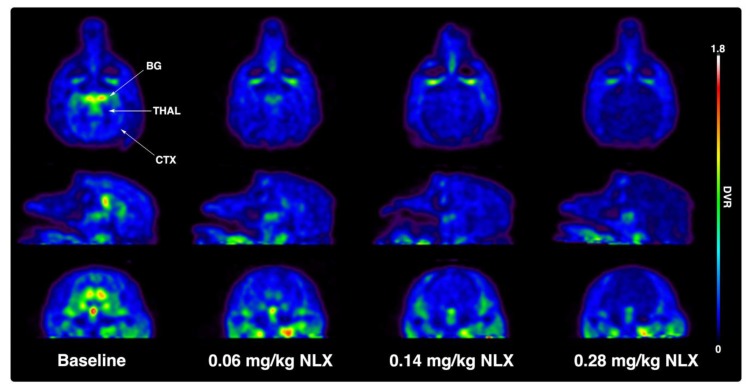
Representative transverse (top row), sagittal (middle row), and coronal (bottom row) monkey positron emission tomography (PET) images of a [^11^C]CFN baseline scan and following blocking studies with low (0.06 mg/kg), medium (0.14 mg/kg), and high (0.28 mg/kg) doses of naloxone (NLX). Images are distribution volume ratio (DVR) images summed 0–60 min following intravenous (IV) injection of the radiotracer. BG = basal ganglia, THAL = thalamus, CTX = cortex.

Initially, two rhesus monkeys received baseline PET scans with [^11^C]CFN (Figure 1 and Table 1). The scans showed the expected distribution of [^11^C]CFN corresponding to the known mu opioid receptor density in the primate brain, with pronounced uptake in the basal ganglia (BG) and thalamus (THAL), as well as moderate uptake throughout the cortex (CTX). Following baselines studies, [^11^C]CFN PET scans were repeated after dosing the monkeys IM with low (0.06 mg/kg), medium (0.14 mg/kg), and high (0.28 mg/kg) doses of NLX (Table 1). To enable comparison of results from this study to existing human data from the literature (see Discussion below), human equivalent IM doses were also estimated from the monkey doses using Ahmad’s approach (Table 2) [14]. The radiotracer was injected 10 min after IM NLX and dose-dependent blockade of [^11^C]CFN by NLX in the BG, THAL, and CTX regions of the monkey brain was apparent (see representative images from one animal in Figure 1).

**Figure 2:**
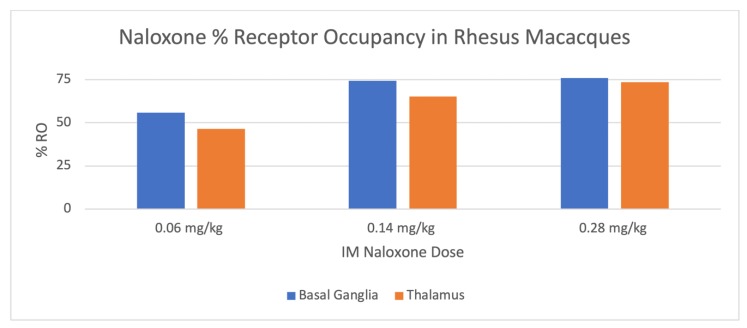
Mean % RO by intramuscular (IM) Dose.

**Figure 3:**
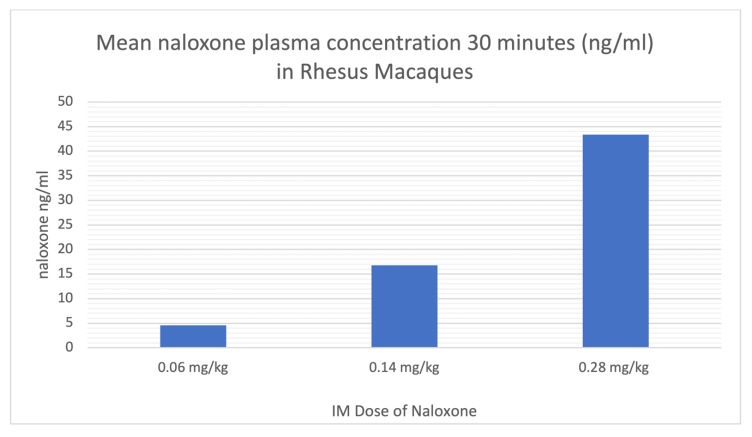
Plasma NLX levels 30 min after three different IM doses.

**Figure 4:**
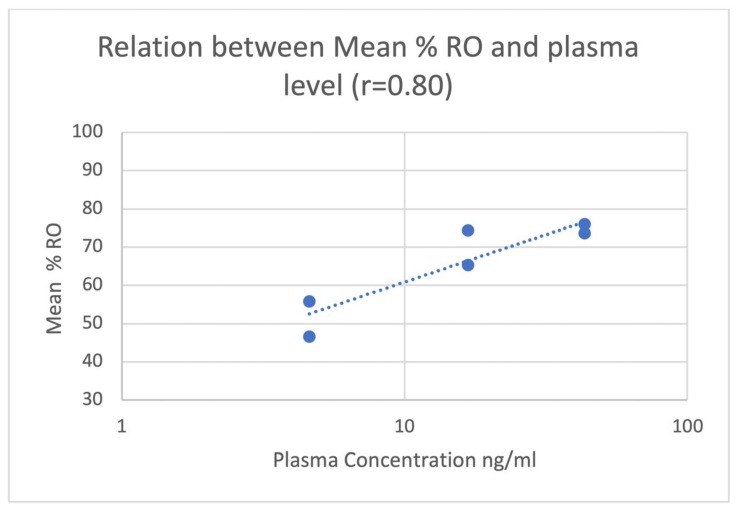
The relationship (r = 0.80) between NLX plasma level and RO in the basal ganglia and thalamus.

The mean % RO by naloxone in the basal ganglia and thalamus is shown in Table 3 and Figure 2. During blocking studies, blood samples were taken from the primates at 0, 30, and 60 min post-injection of naloxone. Plasma was separated and a quantitative LC-MS/MS method was utilized to determine plasma concentrations of NLX, and mean plasma levels 30 and 60 min post-IM injection of the different doses are shown in Table 4 and Figure 3. A significant correlation was observed between plasma NLX concentration and mu RO (r = 0.80) for both the basal ganglia and thalamus (Figure 4).

## 3. Discussion

Previous studies have examined the pharmacokinetics of NLX in different species, as well as various doses and dosage forms. These studies have revealed minimal species differences in, for example, metabolism [15], while establishing that ~50% of the drug is bound to plasma proteins (albumin) and the half-life in plasma is 1–2 h [16]. Prior studies in humans and primates, for example, have examined IV or IN delivery of NLX, including our study comparing the % RO for IN and IV administration in rhesus monkeys. Following a dose of NLX given by two different routes, IV administration resulted in greater RO (75%) compared to the same dose administered by the IN route (65%) [12]. Previous studies of IV administration of NLX in humans suggested that 1 mg IV resulted in approximately 50% occupancy [11]. Other studies in humans suggested that 2 mg IV of NLX resulted in approximately 80% RO [17]. Furthermore, the 2 mg IV dose was associated with a C_max_ plasma level of 38.7 ng/mL. A recent study in humans suggested that 1 mg and 2 mg IV NLX resulted in a mean RO of 54–82% and 71–96%, respectively [18]. The 1 mg and 2 mg IN doses of NLX in that study were associated with C_max_ pharmacokinetic levels of 1.83 ng/mL and 4.33 ng/mL, respectively. Human IM naloxone doses of 0.4, 0.8, and 2 mg have resulted in C_max_ levels of 1.2–1.3, 2.2, and 7.9 nm/mL, respectively [19,20,21]. However, to the best of our knowledge, mu RO following IM NLX has not been investigated in humans or primates. This paper reports the first study in rhesus macaques examining mu RO using [^11^C]carfentanil PET imaging. RO was compared to NLX plasma concentrations quantified using an LC-MS/MS method.

Rhesus monkeys initially received baseline PET scans with [^11^C]CFN (Figure 1 and Table 1). Logan analysis was conducted to determine distribution volume ratio (DVR) [22]. Subsequently, [^11^C]CFN PET scans were repeated 10 min after dosing the monkeys IM with low (0.06 mg/kg), medium (0.14 mg/kg), and high (0.28 mg/kg) doses of NLX. Changes in RO were estimated from the distribution DVR calculated in the baseline scan compared with the DVRs obtained in the NLX blocking studies. Since Logan analysis needs to be conducted after [^11^C] CFN reaches equilibrium (30–40 min post-injection of the radiotracer), RO values determined in this study are averaged for the PET scan but weighted toward 30–60 min post-injection of [^11^C]CFN (40–70 min post-injection of naloxone). At the lowest dose used in this study (0.06 mg/kg), IM NLX averaged 52% RO with a plasma level of 4.6 ng/mL 30 min post-injection, while the 0.14 mg/kg dose resulted in a mean RO by NLX of 70% and a plasma concentration of 16.8 ng/mL. Lastly, the highest dose of IM NLX (0.28 mg/kg) resulted in a mean RO of 75% and a plasma level of 43.4 ng/mL. The plasma levels determined in this study (Table 3) are consistent with known values following IM administration to humans [19,20,21]. It is not always straightforward comparing data between dosage forms (IM/IV), across species (monkey/human), and analytical techniques (imaging/plasma concentrations). However, by extrapolation, the lowest IM dose in this study (0.06 mg/kg) appears to have similar RO and plasma levels to a 1 mg IV dose of NLX in humans [11], which is consistent with the estimated human equivalent dose of 1.2 mg IM (Table 2). Similarly, the highest IM dose of NLX examined in this study (0.28 mg/kg), which corresponded to an estimated dose of 5.4 mg IM to an average 60 kg human (Table 2), appears to result in a comparable RO and plasma level to a 2 mg IV NLX dose in humans [17]. As stated above, it has been previously reported that C_max_ values are lower for IM doses of naloxone than for the equivalent IV doses [23], and our data support these findings.

Most importantly, we observed a direct correlation between the plasma levels of NLX and the RO for IM injections of NLX. Therefore, the plasma level of NLX, which is dependent on the administered dose, appears to be a good predictor of RO. By comparison in humans, the plasma levels associated with the lowest dose of IM NLX in this study are comparable to levels observed with the 4 mg IN and 2 mg IM doses of NLX [10]. Interestingly, the middle-dose group in this study (0.14 mg/kg) was found to have plasma NLX levels comparable to those resulting from a higher 5 mg IM dose of NLX administered to human subjects [7].

A number of factors may affect the competitive binding of NLX to the mu receptor. For example, K_i_ values for mu receptors for both NLX and the opioid-involved are important factors to consider. In addition, we have previously shown in a predictive model that the level of mu receptor-bound opioid impacts the level of NLX needed for opioid toxicity reversal [unpublished results]. In the current macaque model, a subclinical dose of radiolabeled [^11^C]carfentanil was used to estimate RO resulting from an IM injection of naloxone. Thus, the results of this study may underestimate the dose and plasma level of NLX required to achieve a clinically significant level of RO, as very high levels of synthetic opioids have been found in overdose patients [24,25,26,27,28]. However, this study does suggest that for an invariable dose of carfentanil, increasing doses of IM NLX result in greater RO and, furthermore, increasing doses of IM NLX results in higher systemic levels. Lastly, we observed a significant correlation between percent RO and plasma NLX plasma levels and, taken together, these results support the notion that higher doses of IM NLX result in higher mu RO. Given that higher doses of naloxone could be needed to treat overdoses due to more potent synthetic opioids such as fentanyl and carfentanil [8,9], development of higher dose forms of naloxone is warranted in light of the findings in this study.

## 4. Materials and Methods

### 4.1. General Considerations

Primate imaging studies were performed at the University of Michigan in accordance with the standards set by the Institutional Animal Care And Use Committee (IACUC) (Protocol 000008103, Biodistribution and Pharmacokinetics of Radiolabeled Compounds, 1/16/2018–1/16/2021) and all applicable federal, state, local, and institutional laws or guidelines governing animal research. 

### 4.2. Subjects

Two intact, mature female rhesus monkeys were used in this study, aged 19.5 ± 0.7 years and weighing 9.4 ± 0.4 kg, without controlling for the phase of their menstrual cycle. Both monkeys were individually housed in steel cages (83.3 cm high × 76.2 cm wide × 91.4 cm deep) on a 12-h light/12-h dark schedule. Monkeys were fed Laboratory Fiber Plus Monkey Diet (PMI Nutrition International LLC, St. Louis, MO, USA) that was supplemented with fresh fruit daily. Water and enrichment toys were available continuously in the home cage. Each monkey has served in previous PET imaging and blocking studies, corresponding to a fairly extensive drug administration history.

### 4.3. Nonhuman Primate PET Imaging Studies

Primate PET imaging studies were performed using a Concorde Microsystems P4 PET scanner (Siemens, Knoxville, TN, USA). The animals were anesthetized in the home cage with ketamine (Par Sterile Products, Chestnut Ridge, NY, USA) and transported to the PET facility. Subjects were intubated for mechanical ventilation, and anesthesia was continued with isoflurane (Patterson Veterinary Supply Inc., Devens, MA, USA). Anesthesia was maintained throughout the duration of the PET scan. A venous catheter was inserted into one hind limb and the monkey was placed on the PET gantry with its head secured to prevent motion artifacts. Following a transmission scan, IM NLX (or saline in the case of the baseline studies) was administered. Ten minutes later, 3.4 ± 1.4 mCi of [^11^C]CFN was administered in a bolus dose over one minute. Mass of CFN administered was ≤0.03 µg/kg, consistent with our clinical dose limit [13]. Emission data were collected beginning with the injection and continued for 60.0 min (12 × 5-min frames). Data were corrected for attenuation and scatter and reconstructed using the three dimensional–maximum a priori method (3D MAP algorithm).

### 4.4. PET Data Analysis

The dynamic sequence of PET images was summed for the baseline scans and regions-of-interest (ROIs) were drawn manually on multiple planes to obtain volumes-of-interest (VOIs) for the thalamus and basal ganglia of the baseline scan for each monkey. The VOIs were then applied to the full dynamic datasets to obtain the regional tissue time-radioactivity data curve. Images from all subsequent [^11^C]CFN scans following NLX blocking studies were registered to that monkey’s baseline scan, using the NeuroStat package freely available on the internet (https://neurostat.neuro.utah.edu/documents/NEUROSTAT2016.RTF), to allow image data to be extracted from the same set of VOIs. These data were used to construct brain tissue–radioactivity curves that were then analyzed with the method of Logan, with the occipital cortex being the reference region [22]. Changes in receptor occupancy were estimated from the distribution volume ratio (DVR) calculated from each VOI in the baseline scan compared with the DVRs obtained from the NLX blocking studies, using Equation (1).

Occupancy(%) = 100 × (1 − (DVR_block_ − 1)/(DVR_base_ − 1)) (1)

### 4.5. Study Drugs

Naloxone Hydrochloride provided in pre-filled syringes (International Medication Systems Ltd., South El Monte, CA, USA) was utilized for IM injections of the two primates at doses of 0.06 mg/kg, 0.14 mg/kg, and 0.28 mg/kg. Synthesis and quality control testing of [^11^C]carfentanil was conducted as previously described [13]. Briefly, [^11^C]CFN was prepared in a TRACER lab FX_C-Pro_ synthesis module (GE Healthcare, Uppsala, Sweden) fitted with an Agilent Bond Elut C2 cartridge (Agilent Technologies, Santa Clara, CA, USA). [^11^C]MeOTf (~1 Ci) was bubbled into a solution of desmethyl carfentanil TBA salt (0.5 mg) (MilliporeSigma, Burlington, MA, USA, prepared as described in [13]) in ethanol, USP (100 µL) (Akorn, Lake Forest, IL, USA) at room temperature for 3 min. After this time, 1% NH_4_OH (1 mL) (Fisher Scientific, Hampton, NH, USA) was added to the reaction vessel. This crude reaction mixture was further diluted with 1% NH_4_OH (5 mL), and the resulting mixture was passed through the Agilent Bond Elut C2 cartridge to trap [^11^C]CFN. The cartridge was washed with 20% EtOH (3 mL) (Decon Laboratories, King of Prussia, PA, USA), followed by Milli-Q water (10 mL), to remove unreacted precursor and impurities from the cartridge, and dried for 1.0 min with He gas. [^11^C]CFN was eluted with EtOH, USP (0.5 mL) and diluted with Sterile Water for Injection, USP (9.5 mL) (Hospira, Lake Forest, IL). The formulated product was then passed through a Millipore-GV 0.22-µm filter (MilliporeSigma, Burlington, MA, USA) into a sterile dose vial (Jubillant Hollister-Stier, Spokane, WA) and analyzed for pH, radiochemical purity, mass of CFN, and molar activity as previously described [13].

### 4.6. Plasma Naloxone Assay

A quantitative LC-MS/MS method with an internal standard was developed for use in determination of the concentration of NLX in monkey plasma. Specificity, range, and linearity were assessed. For specificity, extracted ion chromatograms of monkey plasma, and monkey plasma spiked with an internal standard, demonstrated that monkey plasma does not interfere with NLX or internal standard quantification. For linearity and range, the analytical calibration curve was constructed with 8 non-zero standards by plotting the peak area ratio of NLX to the internal standard versus the concentration. The concentration range was evaluated from 0.5 to 100 ng/mL for quantification. A blank sample (matrix sample processed without internal standard) was used to exclude contamination or interference. The curve was assessed with weighted linear regression (1/X^2^). The linearity of the relationship between peak area ratio and concentration was demonstrated by the correlation coefficient (r = 0.9974).

## 5. Conclusions

Mu receptor occupancy and plasma concentrations of naloxone following IM delivery of the drug have been investigated in rhesus monkeys. A significant correlation between percent RO and plasma naloxone level was found and confirms that higher doses of IM naloxone result in higher mu RO. These results support the notion that higher doses of IM naloxone may be useful for treatment of opioid overdoses due to potent synthetic opioids.

## Figures and Tables

**Table 1:** Study Metrics.

Monkey Naloxone Dose (mg/kg)	Monkey 1		Monkey 2	
	[^11^C]CFN PET	PK	[^11^C]CFN PET	PK
0.00 (baseline)	n = 1	n = 1	n = 2	n = 1
0.06	n = 2	n = 1	n = 2	n = 1
0.14	n = 2	n = 1	n = 1	n = 1
0.28	n = 1	n = 1	n = 1	n = 1

**Table 2:** Naloxone dosing [14].

Monkey Naloxone IM Dose (mg/kg)	Human Equivalent IM Dose (mg/kg) [14]	IM Dose to Average 60 kg Human (mg)
0.06	0.02	1.2
0.14	0.045	2.7
0.28	0.09	5.4

**Table 3:** Mean % receptor occupancy (RO) by Dose. ^1^

Dose of Naloxone mg/kg	Basal Ganglia Mean %-RO (± SD)	Thalamus Mean %-RO ± SD
0.06	56 ± 17	47 ± 23
0.14	74 ± 7	65 ± 9
0.28	76 ± 3	74 ± 4

^1^ Data is mean ± SD for all studies (at least n = 1 per monkey, as summarized in Table 1).

**Table 4:** Mean plasma concentration of naloxone at 30 and 60 min post-injection. ^1^

Dose of Naloxone mg/kg	Mean Plasma Conc NLX ± SD [Range] (ng/mL) 30 min Post-IM ^1^	Mean Plasma Conc NLX ± SD [Range] (ng/mL) 60 min Post-IM
0.06	4.6 ± 1.9 [3.3–5.9]	2.3 ± 2.4 [0.7–4.0]
0.14	16.8 ± 2.3 [15.1–18.4]	8.1 ± 1.0 [7.4–8.8]
0.28	43.4 ± 19.0 [30.0–56.8]	23.2 ± 1.6 [22.0–24.3]

^1^ Data is mean ± SD [range] for PK studies (n = 1 per monkey, as summarized in Table 1).
